# Experimental Validation of the MRcollar: An MR Compatible Applicator for Deep Heating in the Head and Neck Region

**DOI:** 10.3390/cancers13225617

**Published:** 2021-11-10

**Authors:** Kemal Sumser, Tomas Drizdal, Gennaro G. Bellizzi, Juan A. Hernandez-Tamames, Gerard C. van Rhoon, Margarethus Marius Paulides

**Affiliations:** 1 Department of Radiotherapy, Erasmus Medical Center Cancer Institute, 3015 GD Rotterdam, The Netherlands; tomas.drizdal@fbmi.cvut.cz (T.D.); gennarobellizzi@gmail.com (G.G.B.); g.c.vanrhoon@erasmusmc.nl (G.C.v.R.); m.m.paulides@tue.nl (M.M.P.); 2 Department of Biomedical Technology, Czech Technical University in Prague, nam. Sítna 3105, 272 01 Kladno, Czech Republic; 3 Department of Radiology and Nuclear Medicine, Erasmus Medical Center Cancer Institute, 3015 GD Rotterdam, The Netherlands; j.hernandeztamames@erasmusmc.nl; 4 Department of Electrical Engineering, Eindhoven University of Technology, 5612 AZ Eindhoven, The Netherlands

**Keywords:** hyperthermia, microwave hyperthermia, MR thermometry, MRI guided interventions

## Abstract

**Simple Summary:**

Hyperthermia treatments where tumor tissue is heated to 40–44 °C for 60–90 min can be hampered by a lack of accurate temperature monitoring. To solve this need, we have designed an MR compatible head and neck hyperthermia applicator: the MRcollar. In this work, we experimentally validated the design, heating capabilities and the MR compatibility of the MRcollar. The MRcollar antennas efficiently transfer the power and have low interaction between the antenna elements. The heating and focusing capabilities satisfy the requirements. The MRcollar can operate in an MR scanner and it can acquire higher quality MR images due to the built in receiver elements. The MRcollar has great potential to improve hyperthermia treatment in the head and neck region and it is now ready for in vivo studies.

**Abstract:**

Clinical effectiveness of hyperthermia treatments, in which tumor tissue is artificially heated to 40–44 °C for 60–90 min, can be hampered by a lack of accurate temperature monitoring. The need for noninvasive temperature monitoring in the head and neck region (H&N) and the potential of MR thermometry prompt us to design an MR compatible hyperthermia applicator: the MRcollar. In this work, we validate the design, numerical model, and MR performance of the MRcollar. The MRcollar antennas have low reflection coefficients (<−15 dB) and the intended low interaction between the individual antenna modules (<−32 dB). A 10 °C increase in 3 min was reached in a muscle-equivalent phantom, such that the specifications from the European Society for Hyperthermic Oncology were easily reached. The MRcollar had a minimal effect on MR image quality and a five-fold improvement in SNR was achieved using the integrated coils of the MRcollar, compared to the body coil. The feasibility of using the MRcollar in an MR environment was shown by a synchronous heating experiment. The match between the predicted SAR and measured SAR using MR thermometry satisfied the gamma criteria [distance-to-agreement = 5 mm, dose-difference = 7%]. All experiments combined show that the MRcollar delivers on the needs for MR—hyperthermia in the H&N and is ready for in vivo investigation.

## 1. Introduction

Clinical studies have shown that hyperthermia increases the effectiveness of radiotherapy and chemotherapy for various anatomical sites, without inducing significant side effects [1,2,3,4]. This adjuvant effect of hyperthermia on radiotherapy was shown also for cancers of the head and neck (H&N) [5,6,7,8,9]. In 2007, we developed the HYPERcollar [10] in order to extend the application of hyperthermia from superficial regions to deeper target regions. This device was improved and replaced by the HYPERcollar3D in 2014 [11,12]. These devices were specifically developed for conformal heating of large target regions ranging from 4 to 15 cm of an advanced H&N disease [13]. Placement of invasive temperature catheters in the H&N region is challenging, and intrinsically provides temperature information from only a limited number of discrete measurement points. Non-invasive temperature monitoring by magnetic resonance thermometry (MRT) has shown clinical potential for obtaining detailed 3D temperature maps from the region of interest [14]. Based on our extensive clinical experience with model-based design from the development of the HYPERcollar and HYPERcollar3D, we have designed the world’s-first magnetic resonance (MR) compatible microwave hyperthermia applicator for the H&N: the MRcollar [15]. This novel design is based on a radically new antenna element and array concept. However, it still requires validation of the heating characteristics and MR compatibility before its introduction for studies in volunteers and patients.

A series of dedicated verification measurements of the heating pattern are recommended before introducing a hyperthermia device in clinical practice [16,17,18]. These verification measurements are aimed at improving the translation of hyperthermia treatment planning (HTP) settings into the clinical routine. While quality assurance measurements make HTP a more reliable tool to ensure target conformal electromagnetic field energy delivery, the uncertainties in tissue electromagnetic and thermal properties affect the reliability of the model based predicted temperatures [19,20,21]. Hence, detailed temperature measurements, both in the target region and healthy tissues, are required to assess the true temperatures achieved and to enable dynamic adaptation of the power distribution for maximizing the applied thermal dose. Non-invasive 3D temperature monitoring during the hyperthermia is therefore needed for exploiting the focusing potential and for delivering the optimal thermal dose and hence achieving the full clinical potential of hyperthermia.

Literature indicates that the proton resonance frequency shift method, i.e., the most common MRT method, can provide 3D temperature maps with around 0.5 °C accuracy [22,23,24]. For H&N hyperthermia, several studies [25,26,27] have shown the feasibility of adopting MR compatible applicators; however, to date, none of them has been adopted in clinical practice. Recently, we presented the novel MR compatible applicator for the H&N region: the MRcollar [15] equipped with a novel dual-function applicator concept [28,29]. In this design, novel MR-transparent Yagi-Uda antennas [30] were combined with dielectric reflector modules (DiPRA) to minimize radiofrequency (RF) radiation towards adjacent antenna elements and towards the RF coil of the MR scanner and to minimize the water bolus volume. Furthermore, this new applicator design uses the dual-function applicator concept. These innovative approaches aim to improve the hyperthermia dose delivery, handling of the hyperthermia device, and enable and improve MRI for hyperthermia treatment monitoring. Still, the applicator manufactured based on this design needs to be validated before its introduction in clinical practice.

The aim of this study was to validate the MRcollar design and the numerical model, as well as to validate in MR environment under the light of Medical Devices Directive 93/42/EEC. First, the electromagnetic design of the antennas and the array was validated by reflection and cross-coupling measurements. Second, the achievable heating rate of the device was measured using optical fiber temperature probes inserted into a cylindrical phantom. Third, focus steering capabilities were measured in a muscle-equivalent cylindrical split-phantom by an infrared (IR) camera. Next, the MR imaging performance of the integrated MRcollar coils were compared to the body coil of the MR scanner by measuring Signal-to-noise Ratio (SNR). Lastly, the feasibility of the heating and treatment monitoring in the MR environment were tested. These steps allowed us to experimentally verify the feasibility of using the prototype MRcollar design for MR guided hyperthermia.

## 2. Materials and Methods

### 2.1. The MRcollar Design

**Figure 1:**
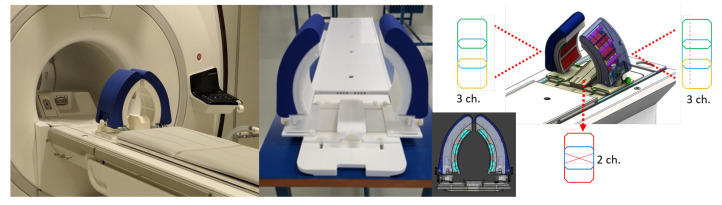
Picture of MRcollar applicator prototype without the inner water bolus and the location of the integrated MR receiver arrays.

The MRcollar (Figure 1) consists of two crescent moon-shaped shells each consisting of six antenna modules in a 2 × 3 arrangement placed around the H&N for 12 antenna setup. Every module contains a printed Yagi-Uda antenna submerged in water and operating at 433.92 MHz. A water bolus, i.e., a “bag” of flexible foil filled with demineralized water, is placed between the antenna modules and the patient surface in order to improve coupling of electromagnetic waves and to prevent unwanted heating of patient’s skin. The MRcollar position can be freely adjusted in all three directions. Furthermore, it can be rotated along the z-axis (sagittal plane) to 15${}^{\circ }$, which is the preferred applicator position for the tumors in the larynx region. The base plate was designed to fit into the dedicated slot on the MR patient bed.

The MRcollar houses 8-channel MR receiver coil array tuned to 63.89 MHz for MR imaging at 1.5 T. Each of the moon-shaped shells include a 3-channel receive coil array (a bottom (15 × 8 cm), a central (15 × 12 cm) and a top (15 × 8 cm) rectangular shaped loops) and the head rest includes a 2-channel receive coil array (butterfly coil 20 × 9 cm and 9 × 9 cm rectangular shaped loops) [29]. The coils are fabricated using etched copper wire. To minimize the coupling between the neighbouring elements, geometric decoupling was used. Decoupling from the transmit coil has been achieved by a passive and active diode detuning circuit.

### 2.2. Phantoms

**Figure 2:**
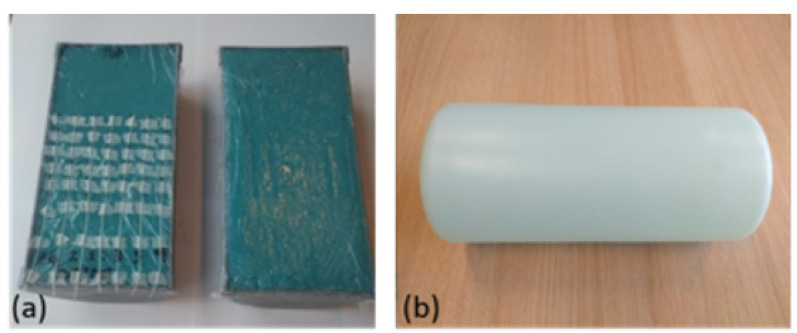
Phantoms (**a**) Cylindrical Split Phantom (**b**) Cylindrical MR phantom.

Two cylindrical “split” phantoms (shown in Figure 2a) were made and filled with muscle-equivalent material consisting of deionized water, agar, sodium chloride, polyethylene powder and TX-151 (electrical conductivity 0.39 S/m, relative permittivity 59, thermal conductivity 0.6 W/m/°C, specific heat capacity 3800 W/kg/°C). Dielectric properties of the phantoms were measured using a dielectric assessment kit (DAK-4, Speag, Zurich, Switzerland). Thermal properties of the phantoms were measured using a thermal property analyzer (TEMPOS, METER Group, Inc., Pullman, WA, USA). The cylindrical phantom (T1: 108 ms, T2: 96 ms; shown in Figure 2b) was provided by the MR vendor (General Electric Health Care, Waukesha, WI, USA).

### 2.3. Antenna Characterization Measurements, Heating and Focusing Steering Capabilities

We measured the S-matrix of the hyperthermia array by attaching a network vector analyzer (ZNC 3, Rhode & Schwarz, Munich, Germany) to the antennas using the cylindrical split phantom as a load.

For the heating characterization measurements, the split phantom was used. MR images of the setup consisting of applicator, water bolus and phantom were acquired before the heating experiment to correctly capture specifically the irregular shape of the water bolus. Then the images were manually segmented using iSeg (v3.10 Zurich MedTech, Zurich, Switzerland). The segmented models of the phantom and the water bolus were manually overlaid with the MRcollar numerical model. Electromagnetic field distributions were computed per antenna channel using Sim4Life (v.4.4.1.3808, Zurich MedTech, Zurich, Switzerland). A 433.92 MHz excitation harmonic signal was applied for 20 periods to each antenna. The size of the calculation domain was 43 million voxels, with a maximum voxel size of 1.5 × 1.5 × 1.5 mm^3^, refined to 0.5 × 0.5 × 0.5 mm${}^{3}$ for the antenna sources. Three heat focus locations were selected, i.e., central focus (0,0,0) mm, x-steered (20,0,0) mm, z-steered (0,0,40) mm, and the corresponding amplitude and phase per channel were calculated using Time-reversal method [31].

The heating experiments were performed by heating the phantom for 180 s with total input power of 180 W. Temperature was monitored during the heating process by 27 fiber optic temperature probes (FISO FOT-NS-577E, Fiso, Quebec, QC, Canada). Specific absorption rate (SAR) was then calculated by the temperature increase in the first 60 s. The temperature distribution of the split phantom was imaged with an IR camera before the heating and quickly (≈10 s after the power was turned off) after opening both halves, after the heating session. The SAR distributions were calculated using the temperature rise method. First, the temperature increase was calculated using the before and after heating IR images. Second, the temperature increase was scaled by the ratio of specific heat ratio of the phantom and the total heating duration to calculate the SAR. Heating focus-size was defined as the length and the width of the 50% iso-SAR contour. The measurements were repeated with an identical phantom. Forwarded amplitude and phase, as well as reflected power, were continuously monitored using the clinical measurement setup [32].

We quantified the match between the measurements and the numerical model using the Gamma-method [33]. We have defined two sets of tolerances: criteria (1) dose difference 10% and distance to agreement 10 mm (gamma10); criteria (2) dose difference 5% and distance to agreement 5 mm (gamma5). The analysis was done using all voxels absorbing at least 30% of the maximum measured SAR in the phantom, to exclude the voxels heated due to thermal conduction.

### 2.4. MR-Compatibility Measurements

The cylindrical phantom was used to evaluate and compare the SNR acquired with the integrated MRcollar coils and the body coil of a 1.5 T MR 450 W scanner (General Electric Health Care, Waukesha, WI, USA). The region where the SNR is calculated shown with a red circle in Figure 5.The evaluation for SNR was done using a fast gradient echo sequence with the following parameters: repetition time 100 ms, echo time 19.2 ms, flip angle 30${}^{\circ }$, number of slices 1, resolution: 1.25 × 1.25 × 3 mm${}^{3}$, matrix resolution 256 × 256, bandwidth 31.2 kHz, number of excitations 1, total acquisition time 25.6 s. SNR was calculated using the dual image subtraction method [34].

The feasibility of using the MRcollar in the MRI environment was tested in a synchronous heating experiment. The cylindrical split phantom was heated for 480 s with total input power of 180 W. Each antenna was excited with equal amplitude and phase. The MRT was calculated using the proton resonance frequency shift method [35]. Images were acquired using a fast gradient echo sequence with the following parameters: repetition time 68 ms, echo time 13.2 ms, flip angle 55${}^{\circ }$, number of slices 3, resolution: 2.5 × 2.5 × 5 mm${}^{3}$, matrix resolution 128 × 128, bandwidth 31.2 kHz, number of excitations, total acquisition time 8.7 s. Approximately 2 min after the heating had been stopped, the split phantom was imaged with an IR camera to validate the MRT results. The MRT slice is 2.5 cm deeper than the split phantom surface where the IR camera measurements were taken.

In Table 1, summary of the measurements which are done for the validation study are given.

## 3. Results

### 3.1. Reflection and Cross-Coupling Measurements

**Figure 3:**
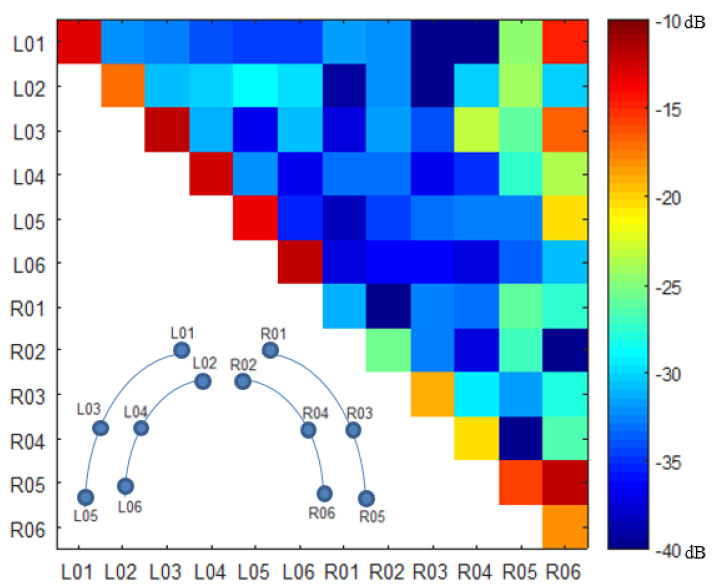
S−matrix and a schematic showing the antenna locations using fish-eye perspective for clarity.

The complete S-matrix of the MRcollar system is shown in Figure 3. The primary reflection coefficient (S${}_{ii}$) of all antennas was on average below the design goal of −15 dB (mean ± standard deviation = −17 ± 6 dB, worst −11 dB) and the cross-coupling between the antennas (S${}_{ij}$) was < −20 dB for all except four combinations (mean ± standard deviation = −32 ± 7 dB, worst −11 dB).

### 3.2. Heating, Steering Capabilities and Focus-Size

**Figure 4:**
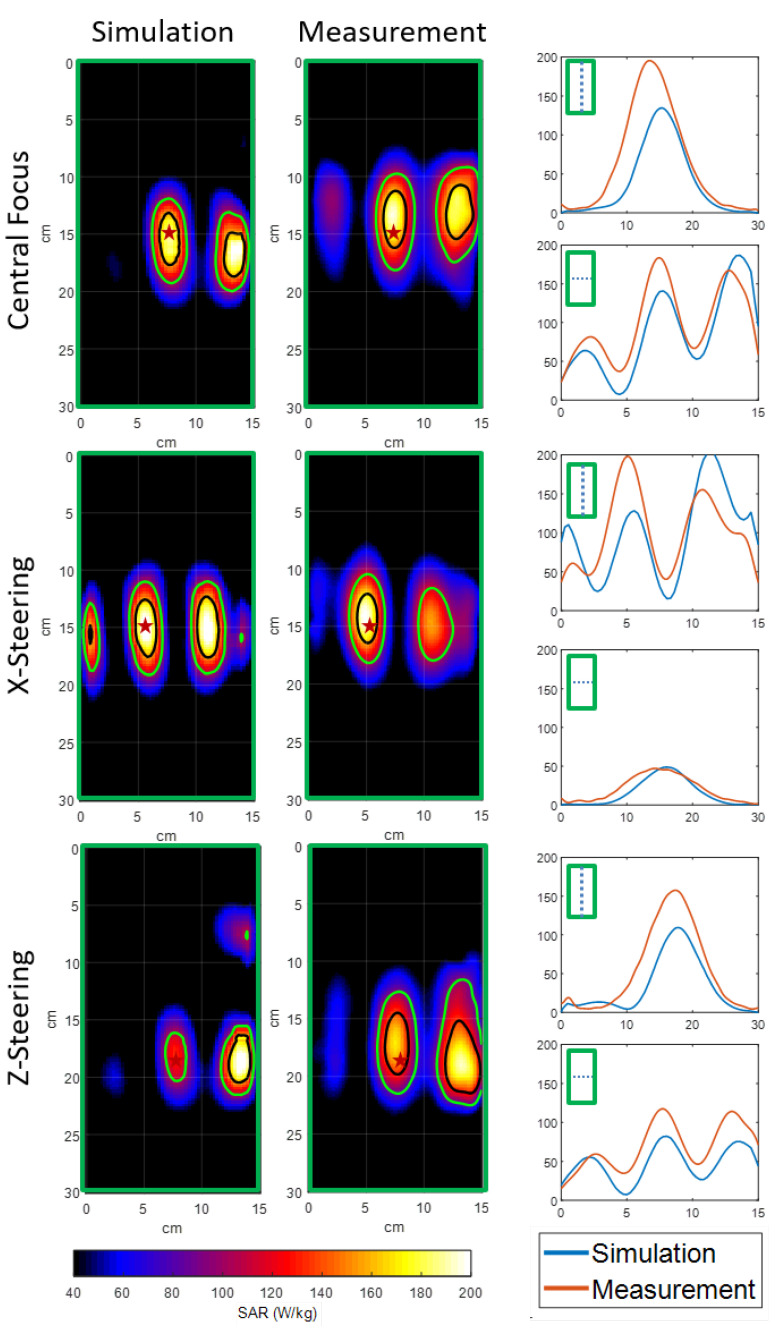
Simulated and measured SAR distributions (**left** and **middle**) and line plot along the phantom’s vertical and the horizontal axis. The green border shows the phantom outline and the red star shows the approximated focus point: central (**top**), x = +2 cm (**middle**) and z = +4 cm (**bottom**).

Using 180 W total input power for 180 s, a 10.3 ± 0.19 °C temperature increase was achieved for all three optimization settings. The maximum measured SAR was 240 and 235 W/kg for central steering, 224 and 221 W/kg for x-steering, and 224 and 227 W/kg for z-steering. The heating patterns for these three different focus locations are shown in Figure 4. The length and width of the focus was 82 and 30 mm for central steering, 85 and 32 mm for z-steering, and 83 and 33 mm for x-steering. The overall results for the gamma analysis is given in Table 2. Gamma10 was full-filled by 88% of the voxels for central steering, 98% for z-steering, and 99% for x-steering. Gamma5 was fulfilled by 72% of the voxels for central steering, 74% for z-steering, and 79% for x-steering.

### 3.3. MR-Compatibility Measurements

**Figure 5:**
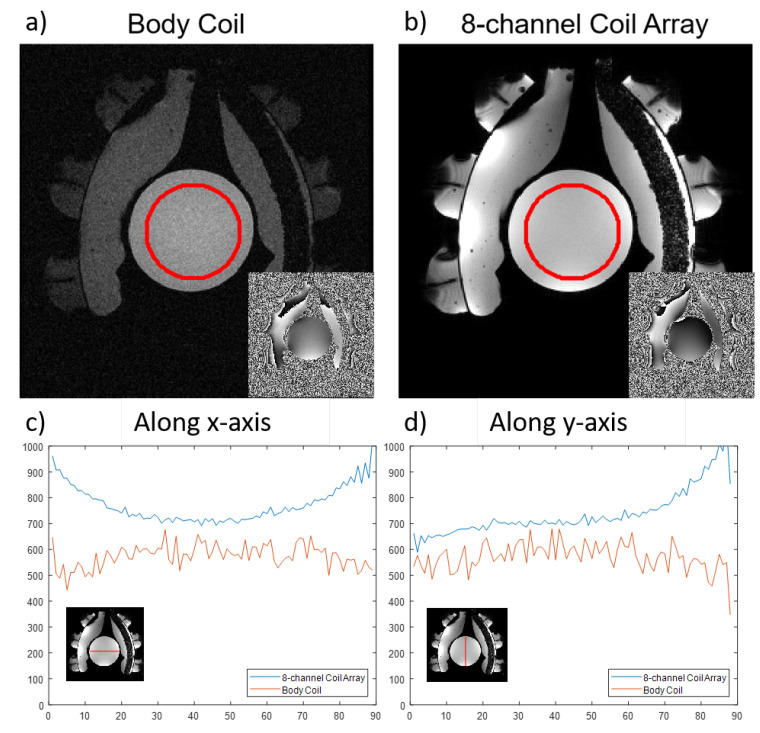
MR magnitude and phase images acquired with (**a**) MR scanner’s body coil (**b**) 8-channel coil array of the MRcollar. The red circle highlights the region of interest where SNR was calculated. Line plots of signal intensities along (**c**) x-axis (**d**) y-axis acquired with the 8-channel coil array (blue) and the body coil (orange).

Figure 5 shows example magnitude images acquired with the body coil and the MRcollar 8-channel receive coil array. The location of the printed antennas are visible as black lines in the DiPRA modules which are filled with demineralized water. Further local MRI distortions are visible in the vicinity of the antennas which are caused by the metallic parts of these printed antenna elements and the connectors. The signal void in the left water bolus is caused by the support structure which is doped with the iron oxide nanoparticles. SNR was 23 for imaging using the body-coil and SNR was improved around five-fold to 120 for the 8-channel coil of the MRcollar.

**Figure 6:**
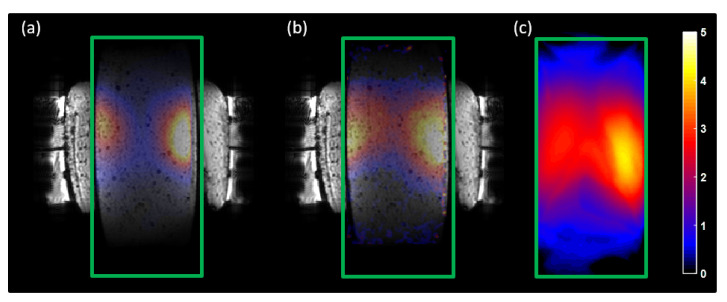
Simulated and measured temperature distributions (**a**) Simulated temperature increase image (**b**) Measured temperature increase with MRT (**c**) Measured temperature increase with IR camera. The green rectangle shows the borders of the phantom.

In Figure 6, the predicted temperature increase (Figure 6a) and measured temperatures (PRFS method: Figure 6b; IR camera: Figure 6c) are shown. The PRFS measurement has a good match with both prediction and IR measurements, showing that the heating ability of the MRcollar is not affected by the MRI environment. Note that the pattern is more spread in the IR measurements due to conduction during the time needed to open the two halves and perform the IR measurement. The gamma match between the prediction and MRT measurements were fulfilled by 99% of the voxels for the gamma10. Gamma5 was passed by 92% of the voxels which does not satisfy the acceptance criteria (95%). The gamma match between the SAR predicted and measured using MR thermometry was satisfied for [distance-to-agreement = 5 mm, dose-difference= 7%].

## 4. Discussion

In this study, we experimentally validated the MRcollar prototype and its numerical model. Antenna loss and inter element cross coupling measurements demonstrated the well matched operation and the very low coupling predicted by earlier simulations [15]. The heating performance predicted by the simulations was validated using the Gamma-method in three phantom experiments. Our results also demonstrate the feasibility and benefit of equipping the MRcollar with receive coils: this greatly improved SNR compared to signal acquisition with the body coil of the MR scanner. The feasibility of using the MRcollar in the MRI environment and the potential of MRT was shown in a synchronous heating experiment. Our results show that hyperthermia treatment in an MR scanner is feasible with the presence of the MRcollar within the MR scanner bore.

### 4.1. EM Compatibility

The DiPRA modules satisfy the original reflection characteristic requirements set for the original single Yagi-Uda antenna by Paulides et al. [30] (S${}_{11}$ < −15 dB) on average. The measured cross coupling between the DiPRA modules showed overall good performance (S${}_{12}$ < −32 ± 7 dB) and matched the predicted performance by simulations (S${}_{12}$ = −27 dB [15]).

### 4.2. Heating Capabilities

The capabilities of the MRcollar prototype to focus and steer the heating pattern were tested using the same tests as those used for the HYPERcollar in Paulides et al. [36]. However, we used the time reversal focusing technique [31] to determine the phase and amplitude coefficients driving the applicator to adjust to the patient conformal shape of the MRcollar prototype and achieve the desired focusing. The measured focus size in the three different configurations (length 83 mm, width 31 mm), is in agreement with the focus size of the HYPERcollar (length 87–112 mm, width 35 mm in [36]) and other applicators operating at 434 MHz [37,38]. The use of the time reversal technique indeed resulted in central and steered foci (with secondary hotspots) so we did not need a more sophisticated optimization approach [39,40,41,42,43,44], which would intrinsically require a target volume and constraints and only blur the focusing in this first validation step. The secondary hotspots which are the product of the time reversal optimization, can be suppressed with addition of constraints to the optimization function.

In the absence of temperature rise criteria for deep hyperthermia applicators, we used those defined in the recent quality assurance document for superficial hyperthermia applicators: at least a 6 °C temperature increase in a maximum of 6 min [17]. The MRcollar fulfilled the above criteria in less than two minutes, i.e., around 70% less than prescribed. Moreover, the total input power used in this study was only 10% of the available power of the clinical amplifiers. Note, however, that the head and neck region is prone to a very high thermoregulation and that smaller heat foci require more energy [45]. Therefore, validation of the true heating capabilities of the MRcollar in patients is still unknown.

The gamma analysis resulted in a good match between the predicted and experimentally measured SAR patterns for all three different focus settings, i.e., 88–99% for gamma10 while they were not satisfactory for gamma5, i.e., 72–79%. In all three cases, the discrepancies were near the edges of the phantom and therefore far from the focus region. The differences can be explained by changes in the shape of the water bag between the experimental setup and the simulated shape. The good match achieved between the predictions and the MRT measurements (99% for gamma10 and 92% for gamma5) also shows that accurate reconstruction of the setup is mandatory to satisfy strict quality assurance criteria. Furthermore, the difficulty in registration of the measurements between the IR camera and the simulations further amplifies the mismatch.

### 4.3. MR Compatibility

Our MR compatibility measurements of the MRcollar show that the metallic parts contained in the antenna structures have a minimal effect on image quality in the phantoms, i.e., the clinically relevant part of the field of view. The susceptibility artefacts induced by the metallic parts were contained in the antenna cavities. Integrating the receiver coils in the MRcollar led to the an important improvement in SNR. The 5-fold increase in the SNR can potentially improve the accuracy and the precision of the MRT results since MRT is a differential measurement method which amplifies the effect of the noise. In addition to the SNR benefits, the integrated coil-array also allows for the use of advanced MR techniques, such as parallel imaging, to enable faster acquisition times (from 8.7 s in this study to 2–4 s) for correction of the impact of respiratory motion. Earlier, we showed that a further increase in SNR and acquisition speed can be achieved by using iron oxide nanoparticles in the water bolus to enable reducing the imaging field of view [46]. All benefits combined can further help the adoption of MR-guided hyperthermia treatments and pave the way for wider acceptance in the clinic.

The heating experiments in the MRI environment proved the feasibility of the dual-function applicator concept. The temperature increase was successfully monitored using the PRFS method and the shape of the predicted and measured temperature increases matched well at the main and secondary hotspots. The near perfect match (99%) was achieved for gamma10 and the result for gamma5 is highly satisfactory (92%). These results also reflect the fact that MR compatibility of a hyperthermia applicator also benefits from having better quality assurance tools. Furthermore, during the treatment, MRI can provide feedback on the patient positioning in addition to the temperature change. The discrepancy in the measured maximum temperature increase between the PRFS and IR camera image can be explained by the two main differences. The IR camera image was taken two minutes after the heating was stopped while the MRT image was acquired as soon as possible. Another issue is that, the visualized slice for the IR camera (center of the split phantom) and the MRT slice (2.5 cm deep from the center) are different.

## 5. Conclusions

In this study, we introduced the MRcollar; the world’s-first MR compatible microwave hyperthermia applicator for the head and neck; and validated its design and operation. Antenna characteristics were measured satisfactorily and the heating rate requirements set by ESHO were satisfied. The measurements and the predicted distributions had a good agreement; satisfying the minimum acceptance criteria set by gamma10. The effect of the metallic structures of the MRcollar on the MRI contained in the local vicinity. The MRcollar receive coils improved the SNR by five-fold, compared to the body coil of the MR scanner. Operation in the MRI environment was shown feasible, and the measured temperature increase distributions matched well to PRFS and IR camera measurements. The conducted experiments on phantoms show that the MRcollar satisfy the requirements for heating and imaging. The MRcollar is now ready for in vivo investigations in the H&N.

## Figures and Tables

**Table 1:** Summary of the measurements.

Topic	Phantom	Measurement Method
Antenna characterization	Cylindrical Split Phantom	Antenna reflection (S${}_{ii}$) and cross coup- ling (S${}_{ij}$) measurements by the VNA.
Heating ability		
SAR${}_{max}$	Cylindrical Split Phantom	Maximum temperature increase in the first 60 s measured by the fiber optic probes and scaled using the specific heat capacity of the phantom as well as heating duration.
Focus Size and steering	Cylindrical Split Phantom	Differential temperature maps from an IR camera before and after 180 s of heating. Focus size was defined as length and width of the 50% iso-SAR contour. This experiment was repeated for three different focus locations with two identical phantoms.
MR compatibility		
MR SNR	Cylindrical Phantom	The dual image subtraction method was performed using a fast gradient echo sequence.
Heating in MRI	Cylindrical Split Phantom	MR images were acquired before and after the 480 s of heating window. Temperature change calculated by PRFS method.

**Table 2:** Results of the gamma analysis.

Variable	Gamma10	Gamma5
Central Focus	88%	72%
X-steering	98%	74%
Z-steering	99%	79%
MRT	99%	92%

## Data Availability

The data presented in this study are available on request from the corresponding author.
